# A Comparative Review of Alternative Fucoidan Extraction Techniques from Seaweed

**DOI:** 10.3390/md23010027

**Published:** 2025-01-07

**Authors:** Matthew Chadwick, Loïc G. Carvalho, Carlos Vanegas, Simone Dimartino

**Affiliations:** 1 Institute for Bioengineering, The School of Engineering, The University of Edinburgh, Edinburgh EH9 3DW, UK; matthew.chadwick@ed.ac.uk; 2 BioMara Ltd., 83 Princes Street, Edinburgh EH2 2ER, UK; loic_decarvalho@biomara.tech (L.G.C.);

**Keywords:** fucoidan, seaweed, extraction, structure, bioactivity

## Abstract

Fucoidan is a sulfated polysaccharide found in brown seaweed. Due to its reported biological activities, including antiviral, antibacterial and anti-inflammatory activities, it has garnered significant attention for potential biomedical applications. However, the direct relationship between fucoidan extracts’ chemical structures and bioactivities is unclear, making it extremely challenging to predict whether an extract will possess a given bioactivity. This relationship is further complicated by a lack of uniformity in the recent literature in terms of the assessment and reporting of extract properties, yield and chemical composition (e.g., sulfate, fucose, uronic acid and monosaccharide contents). These inconsistencies pose significant challenges when directly comparing extraction techniques across studies. This review collected data on extract contents and properties from a selection of available studies. Where information was unavailable directly, efforts were made to extrapolate data. This approach enabled a comprehensive examination of the correlation between extraction techniques and the characteristics of the resulting extracts. A holistic framework is presented for the selection of fucoidan extraction methods, outlining key heuristics to consider when capturing the broader context of a seaweed bioprocess. Future work should focus on developing knowledge within these heuristic categories, such as the creation of technoeconomic models of each extraction process. This framework should allow for a robust extraction selection process that integrates process scale, cost and constraints into decision making. Key quality attributes for biologically active fucoidan are proposed, and areas for future research are identified, such as studies for specific bioactivities aimed at elucidating fucoidan’s mechanism of action. This review also sets out future work required to standardize the reporting of fucoidan extract data. Standardization could positively enhance the quality and depth of data on fucoidan extracts, enabling the relationships between physical, chemical and bioactive properties to be identified. Recommendations on best practices for the production of high-quality fucoidan with desirable yield, characteristics and bioactivity are highlighted.

## 1. Introduction

Fucoidan is a brown seaweed cell wall matrix polysaccharide that makes up approximately 25–30% of brown seaweed’s dry weight [1,2]. Fucoidan’s biological functions are a source of debate [3,4] including, among others, anti-desiccant and antioxidant roles, and it is partly responsible for giving seaweed its mechanical strength and flexibility [5]. Fucoidan possesses a number of bioactive properties, which have been reported to span from antiviral [6] to anti-inflammatory [7], anti-cancer [8] and anticoagulant [9] activities. A comprehensive summary of polysaccharides’ biological effects can be found in Fitton et al. [10]. These bioactivities make fucoidan a molecule of great commercial interest, with the global fucoidan market being valued at USD 30 million in 2022 [11].

Fucoidan is an anionic and water-soluble polysaccharide [12,13,14] mainly composed of fucose monosaccharide units (up to 90%) [15,16]. It can also contain rhamnose, galactose, xylose and mannose at varying concentrations [17,18], plus uronic acids such as galacturonic [19] and glucuronic acids [20]. Two possible structure types are reported in the literature, mainly varying in the way monosaccharide units link with each other, with α-(1-3) links in type 1 or alternating α-(1-3) and α-(1-4) links in type 2 [15,21]. Molecular weights generally range between 10 and 10,000 kDa [19,22,23,24,25], corresponding to about 30 to 6000 monosaccharide units in a single molecule. It is a highly sulfated molecule due to the abundance of sulfate ester groups (6–40%) [26,27], which imbues the molecule with its characteristic negatively charged behavior. It has been suggested that sulfate content [28,29,30,31] and molecular weight [30,32] are key extract characteristics of biologically active fucoidans. However, the exact mechanisms behind these characteristics are a source of debate; this is discussed in depth as part of Section 6.2.

**Figure 1:**
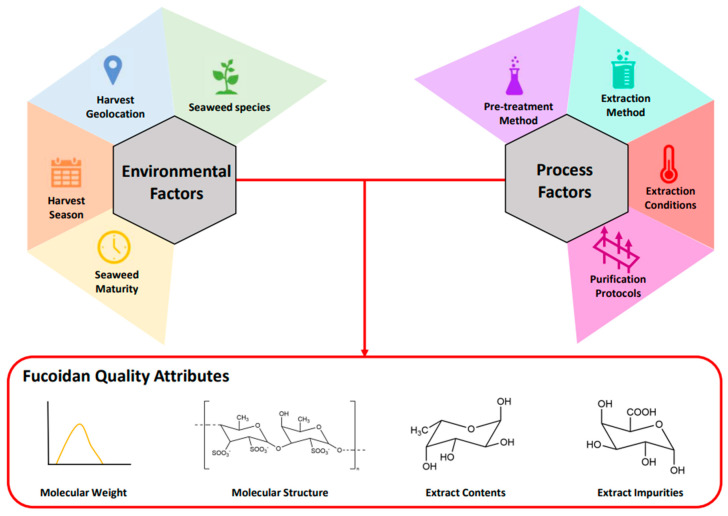
Overview of the biological and process factors affecting fucoidan structure.

The quality of industrial fucoidan products is heavily affected by an incredibly wide range of factors, including biological and environmental aspects, as well as process-related factors (Figure 1).

Factors associated with preprocessing protocols, extraction methods and their operating conditions (e.g., extraction time, temperature, pH, etc.), as well as purification procedures, drastically affect the structure, composition and thus the bioactive properties of extracts [33,34]. However, their precise effects remain unclear.

Moving up the supply chain, biological and environmental factors directly influence the seaweed and the profile of its fucoidan content. The most obvious is variation among seaweed species [30], although similarities are reported among species belonging to the same genus and/or family. For example, fucose content tabulated by Ponce and Stortz widely ranged from as low as 34% for *Ascophyllum nodosum* [35] to well above 90% for various *Fucus* sp. Harvesting location (geographic location) and season can also significantly affect the seaweed polysaccharide content. Geographic location implies specific local environmental factors that the seaweed is exposed to, e.g., shear conditions, water temperature, salinity, nitrogen content and sunlight access [3]. For instance, seaweeds exposed to high shear conditions caused by strong ocean currents tend to adapt to increase their mechanical strength by modulating their polysaccharide profile (e.g., relative quantities of alginate, laminarin and fucoidan) [36]. In this context, Ptak et al. demonstrated a variation in sulfur content of about 25% among fucoidans extracted from *F. vesiculosus* harvested across different geolocations (Denmark and France) [37]. Harvesting season also has a significant effect on seaweed’s polysaccharide characteristics, likely associated with different weather patterns such as solar irradiation and temperature. The same study from Ptak et al. also analyzed fucoidan extracts from *F. vesiculosus*, *F. serratus* and *F. evanescens* harvested in April, July and October, revealing a seasonal difference of up to 86% in sulfur content [37]. On the same note, Fletcher et al. [2] collected samples of *F. vesiculosus*, *F. serratus* and *A. nodosum* every month for a year and analyzed the sulfate and fucose contents of fucoidan extracts. The results highlighted deviations in fucose content between 13.6 and 21.7% and of sulfate content between 14.3 and 59% throughout the seasons. Bruhn et al. suggested that the selection of the correct harvesting time could increase the fucoidan yield by 2 to 2.6 folds [3]. Seaweed maturity has also been demonstrated to considerably affect polysaccharide content, with more mature seaweeds generally having higher fucoidan contents. Multiple explanations have been offered to justify this observation, such as prolonged exposure to free radicals [38] and a higher relative proportion of reproductive tissues [3].

The consistent production of fucoidan products is a key driver of the seaweed-derived product industry. While quality-controlled farmed seaweed can mitigate the influences of biological and environmental factors, the variability in process-related factors remains a major challenge. This variability also makes the comparison of different fucoidan products from different vendors virtually impossible, hindering the application of fucoidan as a bioactive substance in the medical field.

Overall, the relationship between extraction technique, the properties of the resulting fucoidan product and its resultant bioactive properties is poorly established. Jayawardena et al. [39] collected data to study the link between structure and available extraction techniques, with focus on the anti-inflammatory properties of extracted fucoidans. However, they could not find a direct correlation between process variables or extraction techniques and extract contents [39]. Another influential review paper by Mensah et al. covered the literature on extraction techniques and their implications on structure, but it did not provide a link between the extraction conditions or the technique chosen and the resulting fucoidan structure [27]. Luthuli et al. took a different approach by focusing on fucoidan’s biological applications and their links to polysaccharide structure [21]. Unfortunately, the implications between extraction technique, structure and bioactivity are rarely discussed in the literature, as more focus is placed on qualitative discussions around extraction techniques and their advantages and disadvantages.

The goal of this review is to critically assess all relevant information available on fucoidan extraction methods, with particular attention on more recent extraction techniques, and to attempt to draw a link between extraction methods and fucoidan properties. The current literature landscape will be discussed first to place fucoidan extraction procedures into today’s context. Then, the state of the art of fucoidan extraction will be briefly covered, followed by an in-depth analysis of alternative extraction methods, including their relevant operating conditions and resulting extract properties. The entire literature available will then be consolidated to draw out the main trends and characteristics associated with the properties and quality of the fucoidan products obtained with different alternative extraction methods. The paper will conclude with a summary of current challenges and future opportunities to improve fucoidan production in light of the trends uncovered.

## 2. Bioprocessing for Fucoidan Production

**Figure 2:**
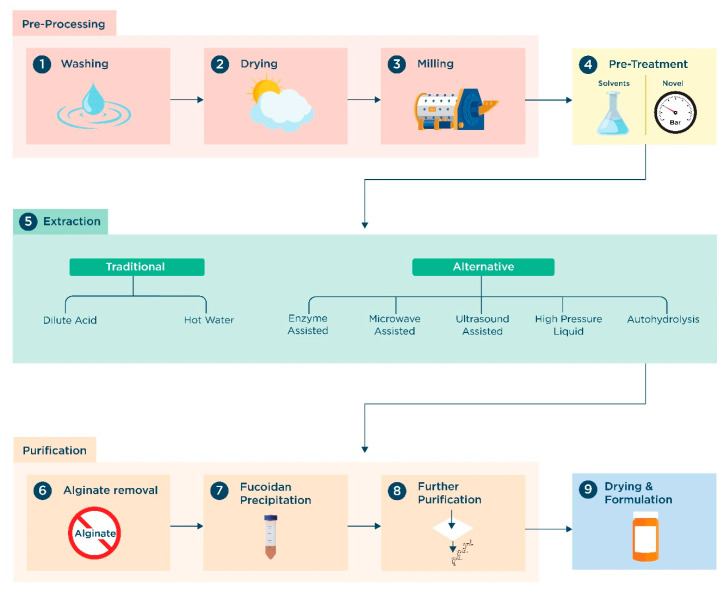
Schematic overview of a typical bioprocess for the production of fucoidans from brown seaweed.

Fucoidan production generally follows a standardized cascade of unit operations (Figure 2), with some differences depending on the extracts’ desired properties and final use. Starting from the harvested seaweed as raw input material, the first stage usually involves a wash to remove dirt and loosen contaminant particulates, after which the seaweed is usually dried to remove excess water, even though this is not a compulsory step, and seaweed can be also processed in a partially dried or wet state. The seaweed is then milled into a powder to increase the surface area-to-volume ratio and increase the efficiency of the extraction procedure.

After mechanical processing, a chemical pre-treatment is usually employed to remove specific classes of impurities and improve the quality of fucoidan extraction [12]. Traditional pre-treatment procedures are solvent-based, in which the milled seaweed is soaked in a solvent such as ethanol, methanol or formaldehyde to remove polyphenols and hydrophobic pigments (mostly chlorophylls) [12,40,41,42,43]. More recently, novel methods such as high hydrostatic pressure and compressional puffing have been developed to lower the solvent and energy requirements of the pre-treatment stage. Both methods create controlled pressure shocks that break apart the cell wall of the milled seaweed, allowing for more efficient impurity removal due to increased mass transfer rates [44,45].

Two main classes of extraction methods are commonly distinguished (Figure 2). Traditional extraction approaches (often termed “conventional”) were developed over 100 years ago and involve soaking seaweed harvests for long periods in harsh conditions, usually in hot solvents at an extreme pH (either acidic or alkaline), to break apart the cell wall and allow for the release of fucoidans. However, these methods are energy intensive and require long extraction times and large amounts of solvents. Alternative methods, often termed “novel” in the literature, have emerged in the last 30 years in the attempt to increase the extraction yield and quality of extracted fucoidans while at the same time reducing the energy and environmental burden associated with conventional extraction. Alternative methods significantly differ in the physical means employed to break the cell wall and include enzyme-assisted extraction (EAE), microwave-assisted extraction (MAE), ultrasound-assisted extraction (UAE) and pressurized liquid extraction (PLE).

Independent of the extraction protocol selected, a complex mixture arises. The main impurities, listed in Table 1, generally arise from co-extracted molecules, chiefly, polysaccharides. The most abundant contaminant is alginate, an extremely abundant polysaccharide accounting for 5–40% of seaweed dry weight [44,46]. Cellulose, hemicellulose and laminarin are other important polysaccharides co-extracted with fucoidan. As well as polysaccharide contaminants, a range of other compounds, including ions such as iodine, small molecules such as phenolics and large proteins, are present.

After extraction, alginate (the most abundant contaminant) is usually precipitated by the addition of calcium chloride, followed by filtration and/or centrifugation to remove the suspended alginate aggregates [50,51,52]. A crude fucoidan can then be precipitated through the addition of ethanol [17,53]. However, to produce a high-quality product, further purification steps, such as ultrafiltration [32,53,54,55] and/or anion-exchange chromatography [53,56], are required. The fucoidan obtained can undergo additional processes, such as spray [57] or freeze drying [58], for formulation into the final product. The cascade of purification units and their operating conditions directly affects the resulting fucoidan product, ultimately impacting its bioactivity.

The extraction and purification techniques employed, together with their operating conditions, have the most significant influence on the final fucoidan product in terms of its composition (e.g., purity and profile of contaminants) and its structure (e.g., branched vs linear structure, molecular weight, degree of sulfation), ultimately influencing the extract’s bioactive properties.

The uronic acid content of a fucoidan extract is often used as an indicator of alginate contamination [22], although this may not be a fair reflection of impurity levels, as fucoidan also naturally contains uronic acids in the form of glucuronic and galacturonic acids. However, the use of a colorimetric assay for the determination of total uronic acids is still widespread in the characterization of fucoidan samples thanks to its simplicity, rapidity and reduced costs [59,60]. More sophisticated analyses able to detect the different monosaccharide types and their contents in a fucoidan sample, such as high-performance anion-exchange chromatography coupled with pulsed amperometric detection (HPAEC-PAD), should be used to obtain a precise fingerprint of the different uronic acids present and better identify contaminant carbohydrates [61].

Fucoidan yield, theoretically defined as the amount of fucoidan in the final product with respect to the fucoidan originally contained in the raw seaweed, is the single most significant parameter employed to judge the performance of fucoidan bioprocessing. Higher yields are generally obtained with harsher extraction conditions, allowing for the recovery of higher proportions of the fucoidans contained in the seaweed cell wall. On the other hand, harsher conditions favor the co-extraction of a range of contaminants, leading to impure fractions that require more complex purification steps, potentially dwarfing the increased yields obtained in the extraction step. More importantly, harsh conditions can degrade the fucoidan either by breaking down the polysaccharide backbone or by removing active groups such as sulfate. As such, it is imperative to holistically consider the entire bioprocessing train for fucoidan production, including the extraction and purification methods employed, as well as their operating conditions.

## 3. Literature Landscape of This Review

In the next sections, focus is placed on alternative techniques due to their reduced energy and solvent consumption compared to traditional methods. A systematic method of data collection from the existing literature was employed. First, a broad Scopus search was conducted using fucoidan and extraction as keywords, followed by filtering to identify all papers that carried out fucoidan extraction using one of the four alternative extraction techniques here considered. Further, papers selected for this review had to directly or indirectly state the extraction conditions employed, as well as provide qualitative or quantitative data of the extracted fucoidan. In instances where data were unclear or missing, reasonable assumptions were made to allow for data to be extrapolated. This approach allowed for the collation of a complete and current data set for each of the four extraction methods. The data collected were tabulated and summarized into boxplots (Section 6.1) to enable direct the comparison and identification of trends across the different extraction methods, with emphasis on alternative techniques. Literature data were standardized to specific descriptors to enable comparison between extraction methods:

- Process properties: yield and purity. Yield was defined as the percentage of crude fucoidan extract collected per unit mass of starting seaweed material. This is a practical definition of yield, as the literature commonly uses total seaweed weight as a reference for this calculation. However, future efforts should be made to define yield relative to an accurately measured fucoidan content in the seaweed source. Fucoidan purity was defined as the mass of fucose obtained over the mass of the total sugar extracted.
- Extract properties: sugar, fucose and sulfate content (per unit mass of extract) and molecular weight (standardized in kDa).
- Extract impurities: uronic acid and phenolic content (per unit mass of extract).

**Figure 3:**
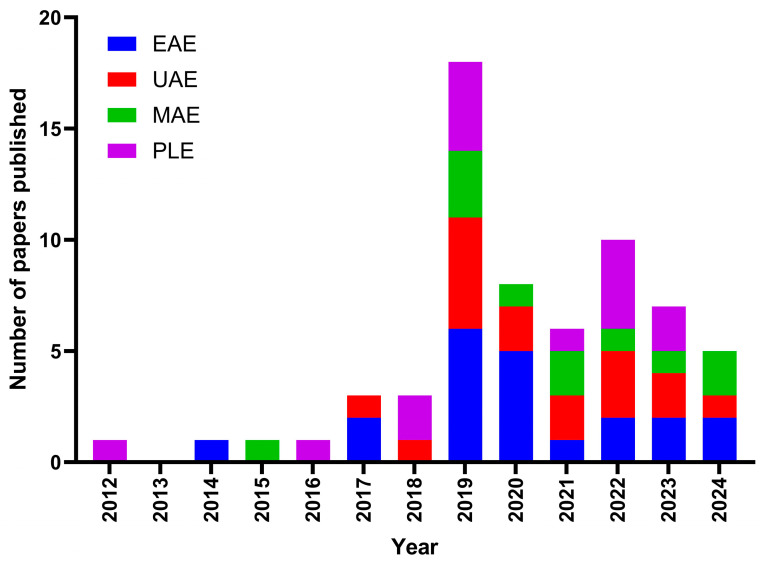
Number of papers published per year that were considered in this review. Enzyme-assisted extraction (EAE), ultrasound-assisted extraction (UAE), microwave-assisted extraction (MAE) and pressurized liquid extraction (PLE).

Figure 3 shows the time distribution of papers considered in this work for the four alternative extraction techniques. The first paper reported is from 2012, with only one or two papers initially published every year, rising to five or more per year from 2019 onward. It is also notable that all extraction methods have received similar interest, with no method clearly dominating over the others, reflecting the current multipronged approach to developing innovative alternative fucoidan extraction techniques.

**Figure 4:**
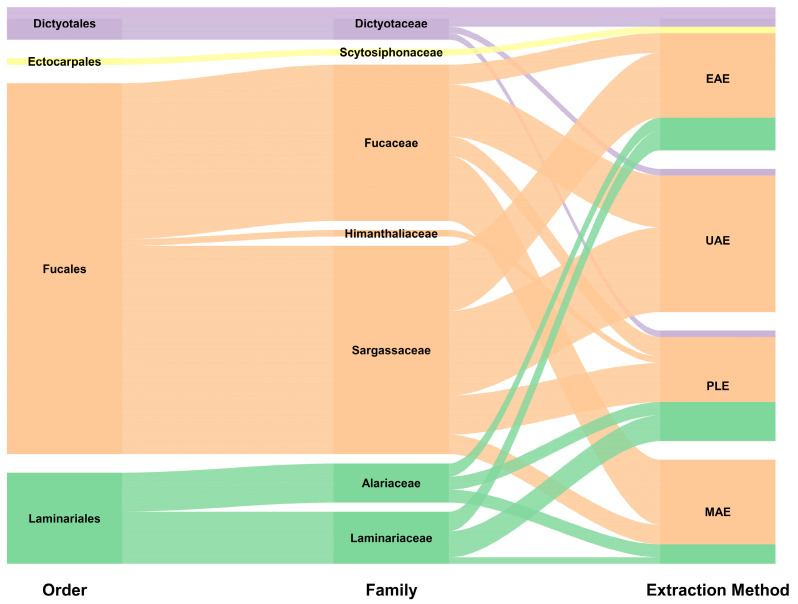
Alluvial diagram showing the taxonomical distribution of seaweed employed in the literature for the various extraction methods: enzyme-assisted extraction (EAE), ultrasound-assisted extraction (UAE), microwave-assisted extraction (MAE) and pressurized liquid extraction (PLE).

Figure 4 depicts the taxonomical distribution of brown seaweeds in the literature selected. The orders Fucales and Laminariales dominate the literature landscape due to a combination of their abundance in nature as well as their relatively high fucoidan content [62]. Over 50% of the seaweed employed in all different extraction methods belongs to Fucales. Within this order, three families are represented, again, with their prevalence closely matching their global distribution: Sargassaceae, followed by Fucaceae and a small quantity of Himanthaliaceae [63,64]. Laminariales species have also been tested using most of the extraction methods except for UAE. Two families represent this order, Laminariaceae and Alariaceae, also aligning with their global prevalence [65]. A small number of papers used kelps from the orders Dictyotales and Ectocarpales. Despite the relatively small presence of Dictyotales and Ectocarpales, they have been tested across the range of alternative extraction techniques. Notably, EAE was applied to virtually all of the taxonomical orders considered. In short, the selected literature provides a globally representative snapshot of the various taxonomical orders and families used for fucoidan extraction. The absence of bias, both in terms of seaweed species considered and in terms of extraction method employed, strengthens the value of the comparisons and conclusions that will follow in this review paper.

## 4. Traditional Extraction Techniques

Traditional extraction methods, hot water extraction and dilute acid extraction, focus on breaking the seaweed cell wall down using high temperatures and/or acidic conditions. These techniques are well established, with hot water extractions dating back to 1915 [66]. Both rely on the fact that fucoidan is a polar and water-soluble macromolecule, for which water acts as an ideal solvent [67]. In hot water extraction, water is added to milled seaweed and heated at temperatures between 60 and 100 °C [68], typically for long extraction times of 1 to 8 h [27,69]. These conditions break down the cell wall and release polysaccharides into solution. After cooling, ethanol is added, causing the fucoidan to precipitate for collection. Dilute acid extraction works on the same principle, with an acidic solvent (generally HCl or H_2_SO_4_ at a pH of 3 to 5) aiding in the breaking of bonds between polysaccharide molecules in the cell wall and enhancing fucoidan extraction efficiency. It employs similar temperatures and extraction times as hot water extraction.

The properties and characteristics of extracts produced using these methods are dependent on four main process variables: temperature, extraction time, pH and solid-to-liquid ratio (typical values between 5 and 100 g of seaweed per L of solvent) [70,71,72].

Typical extract yields vary between 0.1% and 40%, depending on extraction conditions used [72]. Ponce and Stortz collected literature on fucoidan extracts produced from a wide variety of seaweed species using traditional extraction methods [35] and reported a sulfate content of 3% to 38% and uronic acid content of 2% to 29%. Rhein-Knudsen et al. suggested that the molecular weights of extracts vary between 8 and 2400 kDa for hot water and 50 and 100 kDa for acidic extractions [73].

The main advantage of these methods is simplicity [66]. However, the relatively high temperatures employed for prolonged periods of time make these processes particularly energy intensive [67,74]. Also, the harsh conditions used can lead to the degradation of the fucoidan molecules [66]. For example, acidic conditions can hydrolyze the sulfate ester group on the fucoidan molecule, causing a loss of bioactivity [67,75]. Finally, hot water and dilute acid extraction methods are non-selective, leading to high quantities of impurities in the extracts, from uronic acids to polyphenols, limiting the ability to obtain a pure product and requiring increased downstream processing.

## 5. Alternative Extraction Techniques

Traditional methods require long extraction times using large volumes of solvent with high energy demands and low yields in a non-selective manner [76]. These significant drawbacks have led to the development and application of new alternative fucoidan extraction techniques that aim to address some or all of the limitations. This section will first describe their working principles and then discuss the main properties of the extracts obtained in the wider context of the literature selected, concluding with an analysis of their advantages and future opportunities.

### 5.1. Enzyme-Assisted Extraction

Enzyme-assisted extraction (EAE) uses enzymes to lyse the seaweed cell wall. According to this method, the pre-treated seaweed is placed in an aqueous environment in the presence of a mixture of hydrolytic enzymes that catalyze the hydrolysis of the cell wall, releasing its contents [14,71].

Table 2 presents a summary of the literature published on EAE. A notable observation is that the extraction conditions in terms of temperature (40–60 °C), time (24–48 h), pH (4–6) are all broadly the same, corresponding to the optimal conditions for maximum catalytic activity. The characteristics of obtained extracts are also similar, probably due to the same classes of enzyme cocktails being employed.

Non-specific enzyme mixtures containing cellulases and catalases have been mostly tested so far in fucoidan extractions [34,77]. Such enzymatic cocktails were originally developed for plant cells and designed to act on polysaccharides such as starches not found in seaweed [20]. Despite this non-specificity, EAE generally produces higher yields than traditional methods due to more targeted cell wall lysis [78]. The careful optimization of EAE’s operating variables can further increase yields through the adjustment of key extraction variables such as temperature, time, pH, enzyme concentration and the number of extraction stages [79].

Nguyen et al. used cellulase and alginate lyase to break down non-fucoidan polysaccharides in the seaweed cell wall [20]. The effectiveness of the enzymatic procedure was assessed by comparison with traditional extraction methods. The study found that the degree of sulfation of the fucoidan obtained through EAE was around 15% higher than that for hot water extraction, again thanks to the high specificity of enzymatic methods helping to conserve fucoidan’s structure [20].

As shown in Table 2, the molecular weight of extracts produced using this technique tends to be on the order of 100 kDa, likely another result associated with the specific enzymatic cleavage of fucoidan in the cell wall.

Overall, EAE is more sustainable than the traditional method, with significantly lower energy consumption thanks to the lower temperatures required and lower overall solvent consumption [68]. In turn, less solvent usage and lower temperatures lead to reduced hazards associated with the process. Operating costs are further reduced by recovering the enzymes downstream and recycling them for reuse [79]. Perhaps the most obvious disadvantage of enzymatic methods, highlighted in Table 2, is the requirement for much longer processing times (>24 h) in comparison to traditional methods (1–8 h).

**Table 2:** Experimental data on fucoidan extraction using the enzyme-assisted extraction (EAE) technique. Operating conditions are reported in terms of extraction temperature (T), pH, extraction time (t), solid-to-liquid ratio (v_SL_) and solvent used, as well as enzyme type and concentration ([E]). Results are collated in terms of process properties (yield and purity), extract characteristics (sugar, fucose, sulfate contents and molecular weight (MW)) and impurities present (uronic acids and phenolic compounds). Data reported in alphabetical taxonomical order.

Reference	Seaweed Type			Extraction Conditions							Process Properties		Extract Characteristics				Extract Impurities	
	Order	Family	Species	T	pH	t	v_SL_	Solvent	Enzyme	[E]	Yield	Purity	Sugar	Fucose	Sulfate	MW	Uronics	Phenolics
				(°C)		(h)	(g/mL)			(mg/mL)	(%)	(%)	(%)	(%)	(%)	(kDa)	(%)	(%)
Lee et al. (2024) [80]	Dictyotales	Dictyotaceae	*Padina* *arborescens*	50	4.5	24	100	DI H_2_O	Cellulase	10	26.0	41.6	38.9	16.2	5.4	N.D.	N.D.	1.2
Jayawardena et al. (2020) [81]	Dictyotales	Dictyotaceae	*Padina boryana*	50	4.5	24	N.S.	DI H_2_O	Cellulase	N.S.	N.D.	39.8	42.1	16.8 ^b^	4.6	N.D.	N.D.	1.3
Hans et al. (2023) [82]	Dictyotales	Dictyotaceae	*Padina* *tetrastromatica*	50	4.5	24	50	0.1 M NaOAc	Cellulase	50	3.6	N.C.	N.D.	N.C.	11.2	N.D.	N.D.	7.8 ^d^
Fernando et al. (2017) [83]	Ectocarpales	Scytosiphonaceae	*Chnoospora* *minima*	50	4.5	24	10	DI H_2_O	Cellulase	N.S.	N.D.	61.7	68.4 ^e^	42.2 ^be^	28.3	77.5	N.D.	1.0 ^e^
Okolie et al. (2019) [84]	Fucales	Fucaceae	*Ascophyllum nodosum*	50	4.5	24	10	NaOAc	Cellulase	N.S.	3.9	N.C.	N.C.	29.1	15.4	3.9–107.7 ^a^	0.4	N.D.
Deniaud-Bouët (2014) [85]	Fucales	Fucaceae	*Ascophyllum nodosum*	Stage 1:														
				N.S.	7	70	N.S.	0.1 M Tris-MES 0.1 M NaCl 20 mM MgCl_2_	Alginate lyase	N.S.	1.4	N.C.	N.D.	N.C.	N.D.	N.D.	15.3	N.D.
				Stage 2:														
				60	6.5	24	N.S.	0.1 M NaOAc 5 mM EDTA 5 mM Cysteine	Protease	N.S.	18.5	N.C.	N.D.	N.C.	N.D.	N.D.	16.7	1.9
				Stage 3:														
				40	N.S	48	N.S.	0.2% NaN_3_	Cellulase	N.S.	19.2	N.C.	N.D.	N.C.	N.D.	N.D.	12.7	6.4
Nguyen et al. (2020) [20]	Fucales	Fucaceae	*Fucus distichus* subsp. *evanescens* (formerly *F.evanescens*)	40	6	24	20	0.1 M HCl	Cellulase	20	9.9 ^c^	N.C.	N.D.	24.8	21.4	N.D.	72	N.D.
									Alginate lyase	N.C.								
Deniaud-Bouët (2014) [85]	Fucales	Fucaceae	*Fucus distichus* subsp. *evanescens* (formerly *F.evanescens*)	Stage 1:														
				N.S.	7	70	N.S.	0.1 M Tris-MES 0.1 M NaCl 20 mM MgCl_2_	Alginate lyase	N.S.	2.9	N.C.	N.D.	N.C.	N.D.	N.D.	12.1	N.D.
				Stage 2:														
				60	6.5	24	N.S.	0.1 M NaOAc 5 mM EDTA 5 mM Cysteine	Protease	N.S.	15.6	N.C.	N.D.	N.C.	N.D.	N.D.	17.0	10.7
				Stage 3:														
				40	N.S	48	N.S.	0.2% NaN_3_	Cellulase	N.S.	21.4	N.C.	N.D.	N.C.	N.D.	N.D.	25.0	10.7
Deniaud-Bouët (2014) [85]	Fucales	Fucaceae	*Fucus serratus*	Stage 1:														
				N.S.	7	70	N.S.	0.1 M Tris-MES 0.1 M NaCl 20 mM MgCl_2_	Alginate lyase	N.S.	3.2	N.C.	N.D.	N.C.	N.D.	N.D.	12.2	N.D.
				Stage 2:														
				60	6.5	24	N.S.	0.1 M NaOAc 5 mM EDTA 5 mM Cysteine	Protease	N.S.	19.4	N.C.	N.D.	N.C.	N.D.	N.D.	18.4	2.9
				Stage 3:														
				40	N.S	48	N.S.	0.2% NaN_3_	Cellulase	N.S.	31.7	N.C.	N.D.	N.C.	N.D.	N.D.	12.7	34.0
Deniaud-Bouët (2014) [85]	Fucales	Fucaceae	*Pelvetia* *canaliculata*	Stage 1:														
				N.S.	7	70	N.S.	0.1 M Tris-MES 0.1 M NaCl 20 mM MgCl_2_	Alginate lyase	N.S.	1.6	N.C.	N.D.	N.C.	N.D.	N.D.	7.8	N.D.
				Stage 2:														
				60	6.5	24	N.S.	0.1 M NaOAc 5 mM EDTA 5 mM Cysteine	Protease	N.S.	17.4	N.C.	N.D.	N.C.	N.D.	N.D.	11.8	1.7
				Stage 3:														
				40	N.S	48	N.S.	0.2% NaN_3_	Cellulase	N.S.	15.6	N.C.	N.D.	N.C.	N.D.	N.D.	15.0	7.5
Deniaud-Bouët (2014) [85]	Fucales	Himanthaliaceae	*Himanthalia elongata*	Stage 1:														
				N.S.	7	70	N.S.	0.1 M Tris-MES 0.1 M NaCl 20 mM MgCl_2_	Alginate lyase	N.S.	9.9	N.C.	N.D.	N.C.	N.D.	N.D.	16.2	N.D.
				Stage 2:														
				60	6.5	24	N.S.	0.1 M NaOAc 5 mM EDTA 5 mM Cysteine	Protease	N.S.	4.9	N.C.	N.D.	N.C.	N.D.	N.D.	17.0	6.4
				Stage 3:														
				40	N.S.	48	N.S.	0.2% NaN_3_	Cellulase	N.S.	21.9	N.C.	N.D.	N.C.	N.D.	N.D.	N.D.	N.D.
Alboofetileh et al. (2019) [86]	Fucales	Sargassaceae	*Nizamuddinia* *zanardinii*	50	8	24	N.S.	N.S.	Alcalase	N.S.	5.6	32.9	34.7	11.5	20.1	158.9 ^a^	5.7	N.D.
				50	7	24	N.S.	N.S.	Flavourzyme	N.S.	4.4	37.1	36.2	13.4	15.0	127.6 ^a^	13.2	N.D.
				50	4.5	24	N.S.	N.S.	Cellulase	N.S.	4.8	46.8	22.3	10.5	13.6	144.9 ^a^	12.7	N.D.
				50	4.5	24	N.S.	N.S.	Viscozyme	N.S.	4.3	52.1	20.0	10.4	16.4	120.3 ^a^	12.4	N.D.
Alboofetileh et al. (2019) [87]	Fucales	Sargassaceae	*Nizamuddinia* *zanardinii*	50	7	24	N.S.	DI H_2_O	Alcalase	N.S.	5.6	30.8	53.6	16.5	29.6	642.5 ^a^	0.4	N.D.
Alboofetileh et al. (2019) [88]	Fucales	Sargassaceae	*Nizamuddinia* *zanardinii*	50	8	24	N.S.	N.S.	Alcalase	N.S.	5.6	32.9	34.8	11.5	20.1	158.9 ^a^	5.7	N.D.
				50	7	24	N.S.	N.S.	Flavourzyme	N.S.	4.4	37.1	36.2	13.4	15.0	127.6 ^a^	13.2	N.D.
				50	4.5	24	N.S.	N.S.	Cellulase	N.S.	4.8	46.8	22.3	10.5	13.6	144.9 ^a^	12.7	N.D.
				50	4.5	24	N.S.	N.S.	Viscozyme	N.S.	4.3	52.1	20.0	10.4	16.4	120.3 ^a^	12.4	N.D.
Liyanage et al. (2022) [89]	Fucales	Sargassaceae	*Sargassum coreanum*	50	5	8	N.S.	DI H_2_O	Cellulase	5	12.3–1.2 *	N.C.	64.7–46.0 *	N.D.	7.90–20.0 *	50.0–500.0 *	N.D.	0.6–1.4 *
Fernando et al. (2021) [90]	Fucales	Sargassaceae	*Sargassum coreanum*	50	5	8	N.S.	DI H_2_O	Cellulase	5	12.3–1.2 *	N.C.	64.7–46.0 *	N.D.	7.90–20.0 *	50.0–500.0 *	N.D.	0.6–1.4 *
Sanjeewa et al. (2019) [91]	Fucales	Sargassaceae	*Sargassum* *horneri*	50	4.5	24	50	DI H_2_O	Cellulase	N.S.	N.D.	36.9	65.0	24.0 ^b^	12.5	N.C.	N.D.	3.9
Sanjeewa et al. (2017) [92]	Fucales	Sargassaceae	*Sargassum* *horneri*	60	4.5	24	1:50	DI H_2_O	Amyloglucosidase	N.S.	16.0	N.C.	71.6	N.D.	11.5	N.D.	4.6	N.D.
				50	4.5	24	1:50	DI H_2_O	Cellulase	N.S.	20.2	N.C.	88.7	N.D.	12.0	N.D.	3.9	N.D.
				50	4.5	24	1:50	DI H_2_O	Viscozyme	N.S.	21.0	N.C.	74.7	N.D.	11.3	N.D.	3.7	N.D.
				50	8	24	1:50	DI H_2_O	Alcalase	N.S.	22.2	N.C.	81.3	N.D.	2.2	N.D.	3.4	N.D.
Fernando et al. (2020) [93]	Fucales	Sargassaceae	*Sargassum* *polycystum*	50	4.5	24	N.S.	DI H2O	Cellulase	N.S.	N.D.	47.2	70.2 ^e^	33.2 ^e^	23.3 ^e^	N.D.	N.D.	0.4 ^e^
Hans et al. (2023) [82]	Fucales	Sargassaceae	*Turbinaria conoides*	50	4.5	24	50	0.1 M NaOAc	Hemicellulase	50	3.0	N.C.	N.D.	N.C.	14.4	N.D.	N.D.	7.5 ^d^
Jayawardena et al. (2019) [94]	Fucales	Sargassaceae	*Turbinaria conoides*	50	4.5	24	10	1 M HCl	Cellulase	N.S.	N.D.	59.0	71.1 ^e^	42.3 ^e^	23.9 ^e^	N.D.	N.D.	0.3 ^e^
Rhein-Knudsen et al. (2023) [73]	Laminariales	Alariaceae	*Alaria* *esculenta*	55	5.6	24	10	0.025 M NaOAc	Alginate lyase	N.S.	N.C.	N.C.	N.D.	35.0–15.0 ^d^	20.0–42.0 ^d^	N.D.	N.D.	N.D.
Oh et al. (2020) [95]	Laminariales	Alariaceae	*Undaria* *pinnatifida*	50	N.S.	24	20	DI H_2_O	Cellulase	N.S.	6.2	52.3	66.8	34.9	30.4	N.D.	N.D.	N.D.
Tang et al. (2022) [96]	Laminariales	Laminariaceae	*Kjellmaniella* *crassifolia*	50	4.8	10	50	N.S.	Cellulase	4.29	4.7	62.5	76.7	47.9 ^b^	22.8	N.D.	N.D.	N.D.
Rhein-Knudsen et al. (2023) [73]	Laminariales	Laminariaceae	*Saccharina* *latissima*	55	5.6	24	10	0.025 M NaOAc	Cellulase	N.S.	N.C.	N.C.	N.D.	39.0–21.0 ^d^	20.0–45.0 ^d^	N.D.	N.D.	N.D.
Nguyen et al. (2020) [20]	Laminariales	Laminariaceae	*Saccharina* *latissima*	40	6	24	20	0.1 M HCl	Cellulase	20	3.7 ^c^	N.C.	N.D.	12.6	15.5	N.D.	79.5	N.D.
									Alginate lyase	N.C.								
Mabate and Pletschke (2024) [97]	Laminariales	Lessoniaceae	*Ecklonia* *maxima*	50	5	48	30	0.05 M Citrate	Cellulase	N.S.	17.6	N.C.	41.3	N.D.	4.1	5.7	1.2	0.7

N.S. not specified. N.D. not determined. N.C. not possible to calculate. * Determined after a purification step. ^a^ Molecular weight was calculated as the mean of number average and weight average molecular weights. ^b^ Determined from total sugar content by fucose sugar composition. ^c^ Total yield calculated by converting fucose yield using extract fucose composition. ^d^ Value extracted from a graph. ^e^ Value calculated as a weighted average of crude extract fractions.

Numerous opportunities for improvement lie ahead for EAE. The first certainly relates to the development of seaweed-specific enzyme cocktails to overcome the current limitations associated with the use of non-specific enzymes [98]. The selective hydrolysis of the cross-linking bonds of fucoidan with alginate and cellulose to aid in the release of fucoidan from the cell wall not only leads to fucoidan extracts with narrower range of possible molecular weights and higher sulfate contents; it may also lower processing costs due to simplified purification steps [20,85,99]. Further, enzymes could be engineered to tolerate milder conditions, such as even lower temperatures, to provide more process flexibility [85] and/or to lower their costs and enable their use in the processing of large volumes of raw seaweed feeds [98]. For this approach to be successful, the engineered cocktail needs to be compliant with strict regulatory controls on the use of enzymes for the manufacture of food and pharmaceutical ingredients. Another opportunity is represented by enzyme immobilization into solid matrices to avoid the current downstream processing steps for enzyme recycling, with an estimated potential to reduce process costs by around 60% [100,101,102].

### 5.2. Ultrasound-Assisted Extraction

Ultrasound-assisted extraction (UAE) applies ultrasound waves to pre-treated seaweed suspensions, promoting the rupturing of the seaweed cell wall as a consequence of bubble cavitation [14]. This refers to micron-sized bubbles formed within the extraction medium when ultrasound waves are applied. These rapidly collapse soon after formation, creating shock waves that erode the cell wall [103]. Additionally, as a consequence of the turbulence created by these shock waves, seaweed particles collide into each other, breaking apart the cell wall in an attritional process. These two effects combined release the cell wall contents and improve mass transfer to the aqueous phase [104]. For example, Ummat et al. compared the traditional solvent extraction and ultrasound-assisted extraction of phenolic compounds from 11 different species of seaweed. The study found that the yield increased for all 11 species when exposed to ultrasound, with an increase in fucoidan yield of 213% for the species *Fucus distichus* subsp. *evanescens* (formerly *F. evanescens*) and the lowest increase in yield being 46% for *Pelvetia canaliculata* [105].

Table 3 summarizes the published literature on the UAE of fucoidan. As with all methods, there are a multitude of variables affecting the extraction success, including extraction time [68,71,106,107], temperature [68,71,106,107], pressure [68], pH [20,71], solvent and solid-to-liquid ratio [68,107], as well as ultrasound amplitude [106,107] and frequency [108]. A notable observation is the much lower temperatures (25–70 °C) and shorter extraction times (2 to 59 min) in comparison to traditional methods. There is a relatively high variability in ultrasound conditions using typical ultrasound power (150–350 W) and frequency (0.04–40 kHz). A couple of studies used ultrasound methods as pre-treatments before subjecting the seaweed to conventional extraction at 80–85 °C for 2 to 3.5 h.

In general, extraction yields for UAE vary between 3.6% and 33%. These yields are similar to or better than those of traditional methods. Additionally, the molecular weights of extracted fucoidans are in the range of 2.6–1133.9 kDa, slightly misaligned with values in the literature of 10–10,000 kDa [24], with an average value of 20 kDa [19]. This may suggest that additional molecules within the seaweed are also extracted under some extraction conditions, increasing the average molecular weight. This high variability in extract contents and yields may be partially attributed to the use of different species but is more likely a consequence of the numerous differences in extraction conditions employed. This makes it clear that, for this technique to be successful, the extraction conditions must be optimized with the desired product in mind.

It has been suggested that exposure to an ultrasound source increases extraction yields [108]. For example, Hanjabam et al. [66] extracted fucoidan from *Sargassum wightii* by exposure to an ultrasound probe for 30 min at 50% amplitude before precipitating the crude extract with ethanol. The results suggested that the use of ultrasound increased fucoidan yields by 4%. However, the work of Okolie et al. contrasts these findings, where the use of traditional extraction produced a 68% higher yield than standalone ultrasound extraction. It is unlikely that this difference can be solely attributed to external factors such as the seaweed species selected, which in this study was *Ascophyllum nodosum* [84]. Further doubt regarding the suitability of this method was shown by Alboofetileh et al., who tested various extraction methods (enzyme only, ultrasound only, enzyme and ultrasound combined) for *Nizamuddinia zanardinii* seaweed, finding that ultrasound-assisted extraction produced the lowest yield of 3.6%. However, when coupled with enzyme-assisted extraction, it produced the highest yield of 7.9% [87]. Therefore, more work is required in this area to understand what ultrasound conditions lead to greater yields.

The effect of employing ultrasound methods on extract contents remains unclear, as is demonstrated by the range of fucose (0.004 to 0.432), sulfate (0.036 to 0.904) and uronic acid (0.001 to 0.353) (Table 3). For example, Dobrinčić et al. compared extracts from *Fucus virsoides* (*FV*) and *Gongolaria barbata* (*GB*) obtained through traditional and ultrasound-assisted methods. In this work, milled seaweed was suspended in solutions of water, hydrochloric acid or sulfuric acid before being exposed to an ultrasound probe (200 W, 26 kHz, 100% amplitude for 30 min), followed by the precipitation of the extracts using ethanol. The study revealed that extracts obtained using ultrasound methods contained significantly higher sulfate contents (*FV*: 83.4% and *GB*: 90.4%) than those obtained using traditional techniques (*FV*: 28.5% and *GB*: 35.5%). The ultrasound extracts also contained far fewer uronic acids (*FV*: 1.8% and *GB*: 1.2%) compared to traditional methods (*FV*: 20.1% and *GB*: 15.7%). However, there was no clear trend in fucose content, decreasing by 64.5% in *F. virsoides* but increasing in *G. barbata* by 40.7% when ultrasound was applied [109]. On the other hand, Okolie et al. observed that extracts produced using standalone ultrasound extraction techniques had similar fucose and uronic acid contents (27.1%_w/w_ and 0.5%_w/w_) to those obtained using traditional methods (27.4%_w/w_ and 0.6%_w/w_) [84]. Hanjabam et al. found that the fucose content of UAE extracts was 9.5% lower than that of traditional extracts. However, the sulfate contents were broadly similar for both methods [66]. Overall, it is clear that the extraction conditions selected heavily influence the extract contents.

UAE uses reduced extraction times (ranging from two to sixty minutes) [110], as well as a reduced volume of solvents [111], with respect to traditional methods; UAE therefore requires less energy than traditional methods [112], with further energy savings possible when using a pulse mode of operation [113]. A unique additional advantage of UAE is that it can be incorporated alongside traditional or other alternative methods to offer more process flexibility.

A notable disadvantage of this method is the potential depolymerization of the polysaccharide if the treatment time is too long or energy intensive [114,115]. This should be considered when implementing this technology, and the extraction conditions need to be optimized to produce an extract with the necessary chemical fingerprint and an acceptable yield. Further work is required to understand the effects of operating parameters on the resulting extract.

**Table 3:** Experimental data on fucoidan extraction using the ultrasound-assisted extraction (UAE) technique. Operating conditions are reported in terms of extraction temperature, pH, extraction time (t), solid-to-liquid ratio (v_SL_) and solvent used, as well as ultrasound power (P), treatment time (t_u_), current (I), amplitude (A) and frequency (ƒ). Results are collated in terms of process properties (yield and purity), extract characteristics (sugar, fucose, sulfate contents and molecular weight (MW)) and impurities present (uronic acids and phenolic compounds). Data are reported in alphabetical taxonomical order.

Reference	Seaweed Type			Extraction Conditions										Process Properties		Extract Characteristics				Extract Impurities	
	Order	Family	Species	T	pH	t	v_SL_	Solvent	P	t_u_	I	A	ƒ	Yield	Purity	Sugar	Fucose	Sulfate	MW	Uronics	Phenolics
				(°C)	(-)	(Hour)	(g/mL)		(W)	(min)	(A)	(%)	(kHz)	(%)	(%)	(%)	(%)	(%)	(kDa)	(%)	(%)
Hans et al. (2023) [82]	Dictyotales	Dictyotaceae	*Padina* *tetrastromatica*	N.S.	N.S.	N.S.	25	DI H_2_O	N.S.	30	N.S.	50	N.S.	6.2	N.C.	N.D.	N.C.	11.3	N.D.	N.D.	1.0 ^c^
Obluchinskaya and Pozharitskaya (2024) [116]	Fucales	Fucaceae	*Ascophyllum nodosum*	25	4	N.S.	30	5% EtOH	N.S.	N.S.	N.S.	N.S.	22	16.1	N.C.	N.D.	38.1	18.8	364.1 ^d^	0.1102	N.D.
Garcia-Vaquero et al. (2020) [117]	Fucales	Fucaceae	*Ascophyllum nodosum*	N.S.	1	N.S.	10	0.1 M HCl	500	2 or 5	N.S.	20–100	20	N.C.	7.6	2.6	19.5	N.D.	N.D.	N.D.	2.3
Okolie et al. (2019) [90]	Fucales	Fucaceae	*Ascophyllum nodosum*	N.S.	N.S.	N.S.	10	0.01 M HCl	N.S.	35	N.S.	N.S.	20	4.6	N.C.	N.C.	27.1	17.3	2.6–128.7 ^a^	0.5	N.D.
Obluchinskaya and Pozharitskaya (2024) [116]	Fucales	Fucaceae	*Fucus distichus* subsp. *evanescens* (formerly *F.evanescens*)	25	4	N.S.	30	5% EtOH	N.S.	N.S.	N.S.	N.S.	22	17.9	N.C.	N.D.	40.5	22.4	357.0 ^d^	3.6	N.D.
Obluchinskaya and Pozharitskaya (2024) [116]	Fucales	Fucaceae	*Fucus distichus* subsp. *evanescens* (formerly *F.evanescens*)	25	4	N.S.	30	5% EtOH	N.S.	N.S.	N.S.	N.S.	22	21.6	N.C.	N.D.	43.2	24.7	321.2 ^d^	8.6	N.D.
Hmelkov et al. (2018) [118]	Fucales	Fucaceae	*Fucus distichus* subsp. *evanescens* (formerly *F.evanescens*)	N.S.	N.S.	N.S.	20	H_2_O	150	5–30	N.S.	N.S.	35	1.0–3.4	N.C.	N.D.	N.C.	18.1–25.0 ^d^	280.0	N.D.	N.D.
Obluchinskaya and Pozharitskaya (2024) [116]	Fucales	Fucaceae	*Fucus* *serratus*	25	4	N.S.	30	5% EtOH	N.S.	N.S.	N.S.	N.S.	22	15.5	N.C.	N.D.	36.7	0.189	470.9 ^d^	0.0443	N.D.
Dobrinčić et al. (2022) [109]	Fucales	Fucaceae	*Fucus* *virsoides*	N.S.	N.S.	N.S.	N.S.	0.1 M HCl 0.1 M H_2_SO_4_	200	30	N.S.	100	26	12.1	14.8	1.2	0.17 ^b^	83.4	817.0 ^a^	1.8	N.D.
Dobrinčić et al. (2022) [109]	Fucales	Sargassaceae	*Gongolaria* *barbata*	N.S.	N.S.	N.S.	N.S.	0.1 M HCl 0.1 M H_2_SO_4_	200	30	N.S.	100	26	11.8	31.7	0.1	3.8 ^b^	90.4	1133.9 ^a^	1.2	N.D.
Alboofetileh et al. (2019) [107]	Fucales	Sargassaceae	*Nizamuddinia* *zanardinii*	70	N.S.	N.S.	80	DI H_2_O	196	58	N.S.	N.S.	N.S.	3.5	N.C.	N.D.	N.D.	N.D.	N.D.	N.D.	N.D.
Alboofetileh et al. (2019) [87]	Fucales	Sargassaceae	*Nizamuddinia* *zanardinii*	70	N.S.	N.S.	N.S.	DI H_2_O	196	59	N.S.	N.S.	20	3.6	32.3	58.7	19.0 ^b^	23.0	913.5 ^a^	0.1	N.D.
Alboofetileh et al. (2019) [88]	Fucales	Sargassaceae	*Nizamuddinia* *zanardinii*	55	N.S.	N.S.	N.S.	N.S.	200	20 x2	N.S.	N.S.	20	3.6	32.3	58.7	19.0 ^b^	23.0	913.5 ^a^	0.1	N.D.
Laeliocattleya et al. (2023) [119]	Fucales	Sargassaceae	*Sargassum* *aquifolium*	40–60	N.S.	N.S.	20	DI H_2_O	350	10–20	N.S.	N.S.	40	2.8–3.9	N.C.	N.C.	17.2–63.2	5.5–6.2	N.D.	19.1–35.3	1.5–7.8
Thao My et al. (2020) [120]	Fucales	Sargassaceae	*Sargassum mcclurei*	50–56	N.S.	N.S.	24	EtOH	240–480	40–60	N.S.	N.S.	N.S.	33.0	N.C.	N.D.	N.D.	N.D.	N.D.	N.D.	N.D.
Vaamonde-García et al. (2022) [121]	Fucales	Sargassaceae	*Sargassum* *muticum*	25	N.S.	N.S.	N.S.	H_2_O	150	25	1.5	N.S.	0.04	N.D.	38.3	23.0	8.8	3.8	N.C	N.D.	0.2
Del Río et al. (2021) [122]	Fucales	Sargassaceae	*Sargassum* *muticum*	N.S.	N.S.	N.S.	20	DI H_2_O	150	30	N.S.	N.S.	0.04	N.D.	N.C.	N.D.	5.8	3.6	N.D.	N.D.	2.2
Flórez- Fernández et al. (2017) [115]	Fucales	Sargassaceae	*Sargassum* *muticum*	25	N.S.	N.S.	20	N.S.	150	5–30	N.S.	N.S.	0.04	N.D.	N.C.	N.D.	N.D.	4.0	N.D.	N.D.	2.5
Lin et al. (2022) [123]	Fucales	Sargassaceae	*Sargassum* *piluliferum*	Stage 1:										6.3	19.3	28.0	5.4 ^b^	11.5	567.7	14.5	N.D.
				60	6	N.S.	30	DI H_2_O	350	45	N.S.	N.S.	N.S.								
				Stage 2:																	
				80	N.S.	3.5	N.S.	DI H_2_O	N.S.	N.S.	N.S.	N.S.	N.S.								
Wang et al. (2021) [51]	Fucales	Sargassaceae	*Sargassum* *siliquosum*	N.S.	N.S.	N.S.	10	N.S.	50–200	10–20	N.S.	N.S.	N.S.	N.D.	46.6	4.8	2.2	N.D.	N.D.	N.D.	N.D.
Hanjabam et al. (2019) [66]	Fucales	Sargassaceae	*Sargassum wightii*	Stage 1:										14.6	N.C.	N.D.	23.7	17.6	N.D.	N.D.	2.0
				N.S.	1–2	N.S.	25	1 M HCl	N.S.	30	N.S.	50	N.S.								
				Stage 2:																	
				85	1–2	2	25	1 M HCl	N.S.	N.S.	N.S.	N.S.	N.S.								
Hans et al. (2023) [82]	Fucales	Sargassaceae	*Turbinaria* *conoides*	N.S.	N.S.	N.S.	25	DI H_2_O	N.S.	30	N.S.	50	N.S.	8.1	N.C.	N.D.	N.C.	16.4	N.D.	N.D.	N.C.

N.S. not specified. N.D. not determined. N.C. not possible to calculate. ^a^ Molecular weight was calculated as the mean of number average and weight average molecular weights. ^b^ Determined from total sugar content by fucose sugar composition. ^c^ Value extracted from a graph. ^d^ Value calculated as a weighted average of crude extract fractions.

### 5.3. Microwave-Assisted Extraction

In microwave-assisted extraction (MAE), microwave radiation is applied to the pre-treated seaweed suspension. The magnetic field generated by the microwave source induces the movement of dissolved ions as well as the rotation of polar molecules within the cell, leading to localized hot spots [14], breaking apart the cell wall and promoting the extraction of cell wall contents into the solvent [124]. The key processing conditions for MAE include extraction time [125,126], temperature [125,126], solid-to-liquid ratio [125], pressure [125] and microwave power [127].

Table 4 summarizes the existing literature and the variables used. MAE generally uses dilute hydrochloric acid or sulfuric acid as a solvent, with shorter extraction times (1–30 min) compared to those for traditional or enzyme-assisted methods. Typical extraction temperatures range from 60 °C to temperatures exceeding 100 °C. A notable observation is that extractions carried out using species in the Fucaceae family exhibit significantly higher yields (48.5–83.3%) in comparison to those carried out with species from the Sargassaceae (6.2–15.3%) and Alariaceae (6.8–13.7%) families, likely due to differences in the original polysaccharide content of the starting seaweed material. Additionally, fucose (1.7 to 65%), sulfate (0.7 to 48.8%), uronic acid (0.7 to 21.9%) and phenolic (0.4% to 1.0%) contents differ significantly between studies (Table 4).

Dobrinčić et al. carried out extractions of sulfated polysaccharides from two seaweed species, *F. virsoides* and *G. barbata*, employing two extraction methods: traditional (acid) extraction and MAE. The yields obtained through MAE of 20.4% and 15.3% for the two species, respectively, were comparable to the yields obtained with traditional methods of 18.5% and 16.5%, respectively [124]. Okolie et al. studied the extraction of fucoidans from *A. nodosum* using four extraction techniques, including traditional acid and MAE [84]. Traditional extraction led to yields of 11.9%, compared to 5.7% in MAE. The difference between the two studies may be explained by Dobrinčić et al.’s use of an acetone pre-treatment before extraction [124], as well as differences in seaweed species and the selected extraction conditions.

Okolie et al. also found that fucoidan extracts obtained through MAE had a significantly higher uronic acid content (3.59%) compared to those from traditional extraction (0.59%), which could suggest a greater degree of alginate contamination. The molecular weights of the microwave extracts were also significantly lower than those obtained using traditional methods [84]. This is contrasted by Dobrinčić et al., who observed fewer uronic acids in MAE extracts. In addition, a 30% increase in sulfate content, a 17% increase in fucose contents and increased molecular weight and antioxidant capacity [124] were observed.

It is also notable that the purity of extracts varies between studies. For example, Zayed et al. produced extracts with purities ranging from 16% for *Saccharina latissima* to 83% for *Fucus distichus* subsp. *evanescens* (formerly *F. evanescens*), suggesting that the viability of this method is likely dependent on the species employed [127].

MAE is reported to reduce the volume of solvents required, as well as extraction time, by over 75% compared to that for traditional methods [128,129]. Table 4 clearly demonstrates that extraction times for this method are on the order of minutes, with traditional extractions lasting on the order of hours. However, there is no clear evidence in the table of reduced solvent requirements, with similar solid-to-liquid ratios being employed to those for traditional techniques (10–65 g mL^−1^).

MAE reduces operating costs and minimizes process hazards thanks to reduced extraction times. On the other hand, it requires specialist equipment to be implemented at scale, increasing the capital costs of the process.

**Table 4:** Experimental data on fucoidan extraction using the microwave-assisted extraction (MAE) technique. Operating conditions are reported in terms of extraction temperature, extraction time (t), solid-to-liquid ratio (v_SL_) and solvent used, as well as microwave power (P) and time (t_m_). Results are collated in terms of process properties (yield and purity), extract characteristics (sugar, fucose, sulfate contents and molecular weight (MW)) and impurities present (uronic acids and phenolic compounds). Data are reported in alphabetical taxonomical order.

Reference		Seaweed Type		Extraction Conditions					Process Properties		Extract Characteristics				Extract Impurities	
	Order	Family	Species	T	t_m_	P	v_SL_	Solvent	Yield	Purity	Sugar	Fucose	Sulfate	MW	Uronics	Phenolics
				(°C)	(min)	(W)	(g/mL)		(%)	(%)	(%)	(%)	(%)	(kDa)	(%)	(%)
James et al. (2024) [130]	Fucales	Fucaceae	*Ascophyllum nodosum*	90	15	N.S.	20	ChCl-Gly	17.5–22.5	N.C.	N.C.	N.C.	0.7–1.1 ^c^	N.C.	N.C.	0.6–1.0
Garcia-Vaquero et al. (2020) [117]	Fucales	Fucaceae	*Ascophyllum nodosum*	N.S.	2–5	250–1000	N.S.	0.1 M HCl	N.C.	51.2	3.3	1.7	N.D.	N.D.	N.D.	0.1
Okolie et al. (2019) [84]	Fucales	Fucaceae	*Ascophyllum nodosum*	90	15	N.S.	10	0.01 M H_2_SO_4_	5.7	65.8	56.2	3.7	18.8	81.2	3.6	N.D.
Yuan and Macquarrie (2015) [126]	Fucales	Fucaceae	*Ascophyllum nodosum*	90–150	5–30	N.S.	N.S.	0.1 M HCl 0.01 M H_2_SO_4_	6.5–16.1	N.C.	N.C.	N.C.	6.1–28.6	1.3–37.5	N.C.	N.C.
Zayed et al. (2023) [127]	Fucales	Fucaceae	*Fucus distichus* subsp. *evanescens* (formerly *F.evanescens*)	N.S.	1–2	240– 560	10–25	0.1 M HCl	0.9–12.3	83.3	78.0	65.0	48.8 ^d^	16.5	N.D.	N.D.
Ptak et al. (2019) [37]	Fucales	Fucaceae	*Fucus distichus* subsp. *evanescens* (formerly *F.evanescens*)	80–120	30	N.S.	N.S.	0.1 M HCl 0.01 M H_2_SO_4_	3.4–7.8	N.C.	N.D.	N.D.	N.D.	N.D.	N.D.	N.D.
Ptak et al. (2019) [37]	Fucales	Fucaceae	*Fucus serratus*	80–120	30	N.S.	N.S.	0.1 M HCl 0.01 M H_2_SO_4_	4.2–9.5	N.C.	N.D.	N.D.	N.D.	N.D.	N.D.	N.D.
Zayed et al. (2023) [127]	Fucales	Fucaceae	*Fucus spiralis*	N.S.	1–2	240– 560	10–25	0.1 M HCl	N.D.	81.3	64.0	52.0	38.0 ^c^	15.9	N.D.	43.0
Ptak et al. (2019) [37]	Fucales	Fucaceae	*Fucus vesiculosus*	80–120	30	N.S.	N.S.	0.1 M HCl 0.01 M H_2_SO_4_	6.5–11.1	N.C.	N.D.	N.D.	N.D.	N.D.	N.D.	N.D.
Dobrinčić et al. (2021) [124]	Fucales	Fucaceae	*Fucus virsoides*	60–100	N.S.	N.S.	30	0.1 M HCl 0.1 M H_2_SO_4_	20.4	48.5	15.7	7.6 ^c^	37.1	611.7 ^a^	15.9	N.D.
Dobrinčić et al. (2021) [124]	Fucales	Sargassaceae	*Gongolaria barbata*	60–100	N.S.	N.S.	30	0.1 M HCl 0.1 M H_2_SO_4_	15.3	26.6	7.1	1.9 ^b^	45.6	966.8 ^a^	12.5	N.D.
Alboofetileh et al. (2019) [88]	Fucales	Sargassaceae	*Nizamuddinia* *zanardinii*	90	N.S.	700	N.S.	N.S.	6.2	36.3	51.3	18.6 ^c^	24.1	913.9 ^a^	0.7	N.D.
Wang et al. (2021) [51]	Fucales	Sargassaceae	*Sargassum* *siliquosum*	N.S.	N.S.	750	5–25	N.S.	6.9	47.3	33.3	15.8 ^b^	12.2	107.3 *	21.9	6.9
Sasaki et al. (2024) [131]	Laminariales	Alariaceae	*Undaria pinnatifida*	150–170	N.S.	N.S.	67	DI H_2_O	8.8–13.7	N.C.	N.D.	N.C.	6.9–19.2	N.D.	N.D.	N.D.
Vaamonde-García et al. (2022) [121]	Laminariales	Alariaceae	*Undaria pinnatifida*	160	N.S.	N.S.	30	N.S.	N.D.	44.3	25.8	11.4	1.7	N.C.	N.D.	0.4
Zayed et al. (2023) [127]	Laminariales	Laminariaceae	*Saccharina* *latissima*	N.S.	N.S.	240– 560	10–25	0.1 M HCl	N.D.	16.1	56.0	9.0	1.2	18.4	N.D.	11.0

N.S. not specified. N.D. not determined. N.C. not possible to calculate. * Determined after a purification step. ^a^ Molecular weight was calculated as the mean of number average and weight average molecular weights. ^b^ Determined from total sugar content by fucose sugar composition. ^c^ Calculated using sulfation degree and the assumption that each disaccharide unit contains one fucose group, i.e., sulfate content equals sulfation degree multiplied by fucose content. ^d^ Fucose content converted back from the sulfation ratio.

### 5.4. Pressurized Liquid Extraction

In pressurized liquid extraction (PLE), fucoidan is extracted at higher temperatures (>100 °C) and pressures (>>1 atm) than in conventional methods [68]. The high-temperature and -pressure conditions impart the extraction solvents with unique physical properties such as viscosity, density and dielectric constants [132]. Under these conditions, autohydrolysis is promoted, leading to the formation of hydronium (H_3_O^+^) and hydroxy (OH^−^) ions from water molecules [133] and catalyzing the hydrolysis of the seaweed cells, releasing their contents [134]. A unique advantage of PLE is that it offers the flexibility of being operated in a dynamic (continuous) or static (batch) mode [132]. However, the solvent used must be oxygen free to avoid the oxidation of extracts [132]. PLE process variables include temperature, pressure and extraction time, with flow rate as an important parameter for continuous operation [67].

Table 5 summarizes literature results where PLE methods have been used. PLE typically is performed in less than 30 min, with significantly shorter times than traditional techniques. Also, the most common solvent used in PLE is water, offering advantages in terms of disposal and environmental impact. Yields are generally around 20%, comparable with those of other traditional and alternative extraction methods. Saravana et al. carried out both an SWE and a traditional extraction from *Saccharina japonica* [135,136]. The highest crude fucoidan yield, 13.16%, obtained using PL was significantly higher than that for the traditional method (1.8%). This observation was furthered by Dobrinčić et al., who, as mentioned in the previous section, extracted fucoidan using traditional MAE and PLE methods from two seaweed species. The yields of the PLE methods were 24.2% and 18.8%, somewhat higher than those of the conventional methods (18.5% and 16.5%) and MAE methods (20.4% and 15.3%) [124]. Interestingly, much higher sulfate contents of 51.82% and 57.58%, compared to the traditional technique, with sulfate contents of 28.46% and 35.53%, were observed [124]. This is not reflected in Table 5, where average sulfate contents in PLE extracts account for around 20% of the extract.

PLE reduces extraction times [137] and does not require harsh solvents [67,136], leading to reduced operating costs [138], as well as environmental and sustainability advantages. On the other hand, PLE process equipment must be heat, pressure and corrosion resistant, increasing capital costs [67]. Also, the careful optimization of pre-treatment methods and process conditions is required to control extract quality attributes.

**Table 5:** Experimental data on fucoidan extraction using the pressurized liquid extraction (PLE) technique. Operating conditions are reported in terms of extraction temperature, pH, extraction time (t), solid-to-liquid ratio (v_SL_) and solvent used, as well as pressurized liquid pressure (P_i_), treatment time (t_p_) and the number of cycles (n_p_). Results are collated in terms of process properties (yield and purity), extract characteristics (sugar, fucose, sulfate contents and molecular weight (MW)) and impurities present (uronic acids and phenolic compounds). Data are reported in alphabetical taxonomical order.

Reference	Seaweed Type			Extraction Conditions							Process Properties		Extract Characteristics				Extract Impurities	
	Order	Family	Species	T	pH	v_SL_	Solvent	P_i_	t_p_	n_cycles_	Yield	Purity	Sugar	Fucose	Sulfate	MW	Uronics	Phenolics
				(°C)		(g/mL)		(Bar)	(min)	(-)	(%)	(%)	(%)	(%)	(%)	(kDa)	(%)	(%)
Hans et al. (2023) [82]	Dictyotales	Dictyotaceae	*Padina* *tetrastromatica*	150	N.S.	40	DI H_2_O	50	N.S.	N.S.	12.8	N.C.	N.D.	N.C.	13.5	N.D.	N.D.	1.8 ^f^
Getachew et al. (2022) [139]	Fucales	Fucaceae	*Fucus distichus* subsp. *evanescens* (formerly *F.evanescens*)	120–200	N.S.	10	N.S.	N.S.	5	N.S.	4.8–26.0	N.C.	N.D.	2.4–12.5 ^c^	N.D.	N.D.	N.D.	12.1 ^f^
Rodríguez-Jasso et al. (2012) [1]	Fucales	Fucaceae	*Fucus distichus* subsp. *evanescens* (formerly *F.evanescens*)	160–200	N.S.	25	DI H_2_O	N.S.	10–30	N.S.	16.5	N.C.	N.D.	N.C.	18.5–30.8	N.D.	N.D.	3.2–5.4
Dobrinčić et al. (2021) [124]	Fucales	Fucaceae	*Fucus viroides*	60–140	N.S.	N.S.	0.1 M H_2_SO_4_	103.42	10–15	1–2	24.2	60.1	18.2	11.0 ^b^	51.8	335.7 ^a^	5.3	N.D.
Dobrinčić et al. (2021) [124]	Fucales	Sargassaceae	*Gongolaria* *barbata*	60–140	N.S.	N.S.	0.1 M H_2_SO_4_	103.42	10–15	1–2	18.8	28.0	4.4	1.2 ^b^	57.6	723.8	7.2	N.D.
Cernadas et al. (2019) [140]	Fucales	Himanthaliaceae	*Himanthalia elongata*	120–200	4.46–5.3	30	H_2_O	N.S.	N.S.	N.S.	70.7–63.2	23.3	38.9	9.1	2.3–18.3	N.D.	N.D.	0.4–4.6
Alboofetileh et al. (2019) [88]	Fucales	Sargassaceae	*Nizamuddinia zanardinii*	150	N.S.	N.S.	N.S.	N.S.	10	2	13.2	41.7	54.6	22.8 ^b^	11.6	523.2 ^a^	1.9	N.D.
Alboofetileh et al. (2019) [141]	Fucales	Sargassaceae	*Nizamuddinia zanardinii*	90–150	N.S.	N.S.	DI H_2_O	7.5	N.S.	10–30	26.0	34.1	50.5	17.2 ^b^	13.4	694	2.1	N.D.
Huang et al. (2022) [142]	Fucales	Sargassaceae	*Sargassum* *glaucescens*	130–180	N.S.	5–15	DI H_2_O	20–70	15–30	N.S.	N.D.	36.2	1.83	0.066	N.D.	N.D.	N.D.	N.D.
Vaamonde-García et al. (2022) [121]	Fucales	Sargassaceae	*Sargassum* *muticum*	170	N.S.	30	DI H_2_O	N.S.	N.S.	N.S.	N.D.	45.8	64.9	29.7	3.3	N.C.	7.1	3.2
Hans et al. (2023) [82]	Fucales	Sargassaceae	*Turbinaria conoides*	150	N.S.	40	DI H_2_O	50	N.S.	N.S.	150	N.S.	N.S.	40	DI H_2_O	50	N.S.	N.S.
Ferreira-Anta et al. (2023) [143]	Laminariales	Alariaceae	*Undaria* *pinnatifida*	160–220	N.S.	30	DI H_2_O	7.6	N.S.	N.S.	N.D.	N.C.	N.D.	N.C.	1.0–5.0	N.D.	N.C.	0.3–2.5 ^c^
Gan and Baroutian (2022) [144]	Laminariales	Alariaceae	*Undaria* *pinnatifida*	120–210	N.S.	10–30	H_2_O	30	5–30	N.S.	N.D.	N.C.	N.D.	9.0–46.0 ^c^	N.D.	N.D.	N.D.	N.D.
Flórez- Fernández et al. (2019) [145]	Laminariales	Laminariaceae	*Laminaria ochroleuca*	120–200	N.S.	30	H_2_O	7.6	N.S.	N.S.	N.D.	N.C.	N.D.	2.8–17.5 ^c^	26.0–44.0 ^c^	N.D.	N.D.	0.5–2.1 ^c^
Saravana et al. (2018) [146]	Laminariales	Laminariaceae	*Saccharina* *japonica*	100–150	N.S.	30–50	H_2_O	10–15	N.S.	N.S.	5.1–15.7	43.0	43.5	18.7 ^b^	25.7	416.8	15.8	4.8
Saravana et al. (2018) [136]	Laminariales	Laminariaceae	*Saccharina* *japonica*	100–180	N.S.	11–25	0.1% NaOH	20–80	5–15	N.S.	13.6	N.C.	N.D.	48.5	28.6	152.5	14.6	3.5
Saravana et al. (2016) [135]	Laminariales	Laminariaceae	*Saccharina* *japonica*	80–200	N.S.	17	0.1% NaOH 0.1% HCOOH 50–70% EtOH	5–100	5	N.S.	0.1–8.5 ^c^	13.2–100	1.0–31.0 ^c^	1.0–4.1 ^c^	8.0–29.0 ^c^	83.4–216.9	1.5–13.8 ^c^	0.5–3.8 ^c^

N.S. not specified. N.D. not determined. N.C. not possible to calculate. ^a^ Molecular weight was calculated as the mean of number average and weight average molecular weights. ^b^ Determined from total sugar content by fucose sugar composition. ^c^ Value extracted from a graph.

## 6. Comparisons Across Alternative Extraction Techniques

### 6.1. Comparison of Extract Quality Attributes

**Figure 5:**
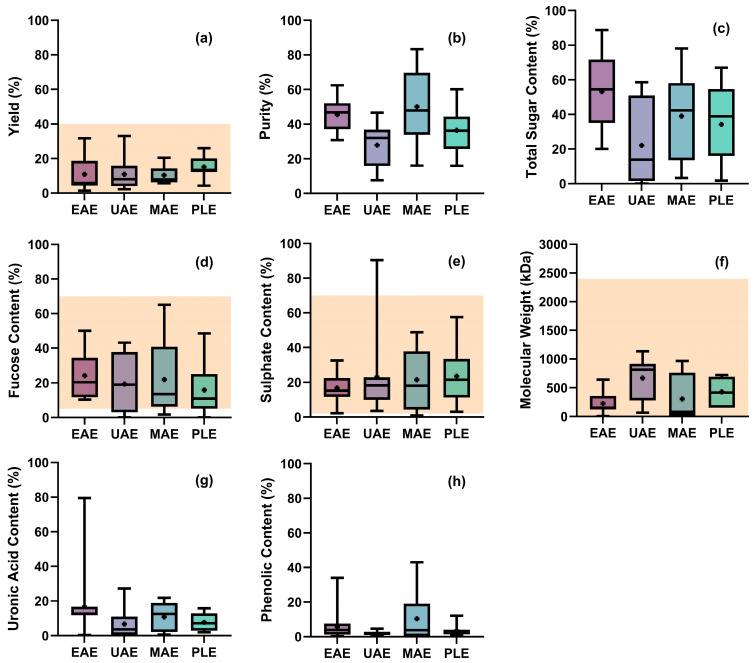
Boxplots of the effect of extraction techniques on fucoidan extract (**a**) yield, (**b**) purity, (**c**) total sugar, (**d**) fucose, (**e**) sulfate content, (**f**) molecular weight, (**g**) uronic acid content and (**h**) phenolic content across the different extraction techniques. The shaded region indicates typical values for traditional extraction methods, a line indicates the median and indicates the mean of each data set.

Figure 5 summarizes data collected on process properties (i.e., yield, extract purity), extract characteristics (i.e., sugar, fucose, sulfate and molecular weight) and impurities (i.e., uronic acid and polyphenols) reported in the previous sections. It is evident that there is a large crossover of process and extract characteristics between methods. Due to the literature’s tendency to benchmark alternative methods against traditional methods, it becomes advantageous to instead focus on making direct comparisons between the alternative methods. These next few sections focus on making comparisons between extraction methods and discussing which characteristics are likely to lead to bioactive extracts.

The data show that fucoidan yields range from 5 to 20%; this falls within the typical range of traditional methods [72], with little variability between the alternative methods (Figure 5a). A similar invariant trend can also be observed for purity (Figure 5b), as well as sugar (Figure 5c), fucose (Figure 5d), sulfate (Figure 5e) and phenolic (Figure 5h) contents.

Figure 5f shows a narrower range of extract molecular weights for EAE. This is likely due to the same cellulolytic or protease-based enzyme cocktails being employed, with similar hydrolytic properties for breaking apart the cell wall in a non-seaweed-specific manner. This leads to extracts with similar molecular weights. A wider range of molecular weights is seen for UAE and MAE methods, suggesting that these methods may require careful control to achieve a desired molecular weight. All of the alternative methods yield molecular weights falling within the same range achieved through the application of traditional extraction methods, suggesting that these methods are comparable using this metric.

Figure 5g shows that the uronic acid content across all methods is comparable, likely due to alginates being removed using calcium carbonate. It is clear that all fucoidan extraction methods can produce fucoidan with similar yields, contents and quality attributes.

To aid in the selection of an extraction method, a holistic approach is required. To achieve this, the following heuristics are proposed and should be considered during process selection. This will allow for the capture of the broader context on a case-by-case basis and aid in the selection of an extraction process.

1. Process Scale: The amount of fucoidan product to be produced must be considered. This is dependent on the amount of raw seaweed material available, as well as the usage of the final product. Traditional methods easily lend themselves to large scales using traditional stirred-tank reactors. However, EAE methods may become more challenging at scale due to a larger demand for enzymes.
2. Operational Mode: It is also important to consider if a batch, semi-continuous or continuous process is most suitable for the production of fucoidan. The use of a continuous process increases production rates and reduces labor costs but does not lend itself to process customization or quality control monitoring between stages like a batch process. As already established, PLE methods have the flexibility to operate in all three of these process types, while EAE methods are limited to batch or semi-batch processes due to their long processing times (~24 h).
3. Economics: Both the capital (CAPEX) and operating (OPEX) expenses associated with the extraction and downstream requirements must be quantified as part of a technoeconomic assessment during process design and selection. Models of the energy and solvent requirements of each extraction technique at different production scales need to be developed in future.
4. Environmental Friendliness and Sustainability: The waste streams present in the process and the impact of their disposal routes should be examined closely. Wherever possible, waste streams from fucoidan production should be valorized and a zero-waste biorefinery approach applied.
5. Regulatory and Safety Constraints: Each extraction method has its own unique regulatory requirements. For example, EAE methods may be constrained regarding the use of enzymes, and PLE methods will require additional controls for safe operation.

Having considered each of these factors and selected an extraction technique, focus should move toward producing an extract with desirable key quality attributes (e.g., sulfate and fucose contents, molecular weight, etc.) at a sufficient yield and purity. To this end, further work is required to understand the relationships between fucoidan extract contents, structure and yields with changing seaweed species, pre-treatment methods, extraction techniques and conditions selected. This would identify relevant extraction conditions for optimization. To identify these, the standardization of key quality attributes becomes essential to drive alternative methods into industrial practice.

### 6.2. Comparison of Extract Bioactivities

Fucoidans possess a wide range of biological activities, including antiviral, antifungal, anti-inflammatory, anti-cancer and anticoagulant properties. As introduced in this review (Figure 1), the quality of the fucoidan is influenced by biological factors (i.e., seaweed species, harvest location, geolocation), as well as the extraction technique and the conditions employed. Changes in these factors will result in variability in fucoidan contents and structural conformation, having a direct effect on fucoidan extracts’ bioactivity. As such, there have been many studies focused on fucoidan as a bioactive substance. Table 6 presents a selection of these studies across a range of biological activities.

The table is demonstrative of the wide range of biological activities that fucoidans can possess. It is also evident that the starting seaweed material and extraction method directly influence biological activity. The antioxidant study carried out by Dobrinčić et al., highlights this, where extracts produced using different species and traditional, MAE or PLE methods all possess different antioxidant properties, confirming that the method and seaweed species have a significant influence on extract bioactivity [124]. Okolie et al. validate this observation through their prebiotic studies, in which different growth rates are observed for different extracts [84]. A study by Alboofetileh et al. furthers this, where an antiviral response is seen in extracts produced using all methods but PLE. Notably, this study also reports that extracts produced through alternative methods outperform traditional extracts, presenting a possible opportunity [88].

What remains unclear is the relationship between biological activity and extract quality attributes. This prompts further investigation to understand the underlying biological processes and mechanisms of action of fucoidan for a given biological activity, as well as the extraction conditions that lead to a biologically active extract. To this end, research has been focused primarily on the effects of fucoidan molecular weight and sulfate contents on biological activity.

A suggested benefit of low-molecular-weight fucoidans is that they possess increased bioavailability. Tan et al. demonstrated this by studying tissue absorption and pharmacokinetics in ICR mice. Their results showed that low-molecular-weight fucoidans were absorbed faster and more efficiently in comparison to high-molecular-weight fractions [147]. As such, studies have been carried out to assess the effects of this extract quality attribute.

Chen et al. found that low-molecular-weight fucoidans enhanced anti-lipogenesis, antioxidant and anti-inflammatory activities, suggesting that bioactivity may be improved with a reduction in molecular weight [31]. Krylova carried out antiviral studies on extracts of differing molecular weights, finding that the low-molecular-weight fractions had more potent antiviral effects [148]. In both studies, the interpretation of these results should be cautious, given the fact that high- and low-molecular-weight fractions in these studies were prepared using enzymatic digestion, which may have altered the fucoidan structure away from something obtainable using solely fucoidan extraction methods. More confidence in these findings is provided by Wang et al., who prepared low- and high-molecular-weight extracts using molecular weight cut-off membranes, finding that the low-molecular-weight fraction induced a greater anti-inflammatory response [149].

**Table 6:** A selection of studies investigating fucoidan bioactivity: antiviral, antioxidant, antibacterial, anti-inflammatory, probiotic and anti-lipogenesis effects.

Reference	Seaweed Species	Microbe(s) Used	Extraction/Preparation Method	Assay	Key Results
Antiviral					
Mandal et al. (2007) [150]	*Polycladia indica (formerly* *Cystoseira indica)*	Vero cells Herpes simplex viruses (HSV1 and HSV2)	Crude fucoidan purified using anion-exchange chromatography, both crude and purified, chemically desulfated.	MTT assay used to determine fucoidan cytotoxic effect. Antiviral activity assessed using a plaque-based assay.	No cytotoxicity was observed in any of the four fractions. Desulfated samples had reduced potency or no antiviral effect against HSV-1 and HSV-2.
Thuy et al. (2014) [151]	*Sargassum mcclurei* *Sargassum* *polycystum* *Turbinaria ornata*	U373-CD4-CXCR4 cellsHIV-1	Crude fucoidan from three species was tested. The sulfate content of two of the crude extracts was increased by anion-exchange chromatography.	Cytotoxic effect determined using LIVE/DEAD staining. Antiviral effect quantified using luciferase relative luminescence measurement.	Cell viability remained between 100 and 60% for all three crude samples. Increased antiviral effects were observed with increasing sulfate content when comparing crude and purified samples.
Alboofetileh et al. (2019) [88]	*Nizamuddinia zanardinii*	Vero cells Herpes simplex virus (HSV2)	Traditional, EAE, UAE, MAE, PLE and combined ultrasound–microwave (UMAE) and enzyme–ultrasound (EUAE) methods used.	MTS assay used to determine fucoidan cytotoxic effects. Antiviral activity assessed using a plaque assay.	No cytotoxic effects were observed. All extracts possessed antiviral properties compared to the positive control. The traditional extract was the most potent antiviral agent, with alternative (UAE, MAE, UMAE and PLE) methods producing a reduced potency.
Krylova et al. (2021) [148]	*Fucus distichus* subsp. *evanescens* (formerly *F. evanescens*)	Vero cells Amur virus	Fucoidan enzymatically digested into high- (FeHMP) and low-molecular-weight (FeLMP) fractions for testing.	MTT assay used to determine fucoidan cytotoxic effects. Antiviral activity assessed using a plaque-based assay.	Low cytotoxicity was found for all fucoidan fractions (>2000 µg/mL). A reduced IC50 value was observed for the FeLMP compared to the FeHMP, suggesting a greater antiviral effect with reduced molecular weight.
Antioxidant					
Yuan and Macquarrie (2015) [126]	*Ascophyllum nodosum*	N/A	Fucoidan with differing molecular weights and sulfate contents produced using different MAE conditions.	Antioxidant capacity assessed using DPPH and reducing power assays.	Antioxidant capacity by both assays was correlated with increasing molecular weight and sulfate content.
Chen et al. (2021) [31]	*Sargassum* *siliquosum*	N/A	Fucoidan produced by MAE method and cut-off membrane purification. Highly sulfated form prepared by chemical modification. Varying-molecular-weight samples produced by hydrogen peroxide digestion.	Antioxidant capacity assessed using a DPPH assay.	The study found that the antioxidant capacity of extracts was inversely proportional to molecular weight.
Dobrinčić et al. (2021) [124]	*Gongolaria barbata* *Fucus virsoides*	N/A	Fucoidans produced using traditional, MAE and PLE methods.	Antioxidant capacity assessed using ORAC and DPPH assays.	Antioxidant capacity varied between methods and species, suggesting that these have an effect on this biological activity.
Wang et al. (2022) [149]	*Ascophyllum nodosum*	RAW264.7 cells	Fucoidan produced by the EAE method and two extracts of differing molecular weights prepared using cut-off membranes.	Production of seven cytokines (inflammation) monitored using PCR. Nitric oxide levels determined using an assay kit.	Both fractions suppressed nitric oxide production and regulated pro-inflammatory cytokine production. The low-molecular-weight fucoidan was a more potent anti-inflammatory agent than the high-molecular-weight extract.
Husni et al. (2022) [152]	*Sargassum hystrix*	N/A	Extracts produced using traditional extraction methods with four different conditions.	Antioxidant capacity assessed using DPPH, FRAP, HRSA and a vitamin C-based assay.	FRAP results suggested a directly proportional relationship between antioxidant capacity and sulfate content. This relationship was not observed in other antioxidant assays because of interference with other compounds in the extracts.
Antibacterial					
Alboofetileh et al. (2019) [88]	*Nizamuddinia zanardinii*	*Escherichia coli* *Pseudomonas aeruginosa* *Listeria monocytogenes* *Staphylococcus aureus*	Traditional, EAE, UAE, MAE, PLE and combined ultrasound–microwave (UMAE) and enzyme–ultrasound (EUAE) methods used.	Disk diffusion method at 2 mg/mL used and quantified using minimum inhibitory concentration (MIC).	PLE (MIC: 1.8 mg/mL) and MAE (MIC: 1.7 mg/mL) inhibited the growth of E. coli. PLE (2 mg/mL), EUAE (2 mg/mL) and UMAE (1.8 mg/mL) fucoidans inhibited the growth of P. aeruginosa. No antibacterial effect was observed on L. monocytogenes or S. aureus for any extraction method.
Anti-inflammatory					
Chen et al. (2021) [31]	*Sargassum siliquosum*	RAW264.7 cells	Fucoidan produced by the MAE method and cut-off membrane purification. Highly sulfated form prepared by chemical modification. Varying-molecular-weight samples produced by hydrogen peroxide digestion.	Pro-inflammatory cytokine levels (TNF-α) determined by ELISA	The level of TNF-α production (inflammation) decreased with increased sulfate content and a reduction in molecular weight.
Wang et al. (2022) [149]	*Ascophyllum nodosum*	RAW264.7 cells	Fucoidan produced by EAE method and two extracts of differing molecular weights prepared using cut-off membranes.	Production of seven cytokines (inflammation) monitored using PCR. Nitric oxide levels determined using an assay kit.	Both fractions suppressed nitric oxide production and regulated pro-inflammatory cytokine production. The low-molecular-weight fucoidan was a more potent anti-inflammatory agent than the high-molecular-weight extract.
Prebiotic					
Okolie et al. (2019) [84]	*Ascophyllum nodosum*	*Lactobacillus delbruecki* subsp. *bulgaricus* *Lactobacillus casei*	Fucoidans produced using traditional, EAE, MAE and UAE methods.	Growth rate determined using an optical density growth curve assay for 24 h.	All fucoidans improved the growth of *L. delbruecki ss bulgaricus* in a statistically significant way. No such trend was seen for *L. casei.*
Anti-lipogenesis					
Chen et al. (2021) [31]	*Sargassum* *siliquosum*	HepG2 cells	Fucoidan produced by MAE method and cut-off membrane purification. Highly sulfated form prepared by chemical modification. Varying-molecular-weight samples produced by hydrogen peroxide digestion.	Lipid formation determined using an oil red staining assay.	With increased sulfate content, the anti-lipogenesis activity decreased. Anti-lipogenesis activity increased with decreasing molecular weight. A low-sulfate-content, low-molecular-weight sample may be favorable for a high anti-lipogenesis activity.

However, studies such as that by Yuan and Macquarrie counter this finding, showing that the reducing power of extracts increased with molecular weight [126]. As such, it may be the case that different ranges of molecular weights are required for different biological activities. Due to this discrepancy in the literature, it may be preferable to produce and refine fucoidan extracts into a pure, high-molecular-weight form prior to digestion into low-molecular-weight fractions and formulation into final products for specific bioactivity applications.

Sulfate content has also been suggested to influence fucoidan biological activity. Husni et al. produced fucoidan using four extraction methods before testing their antioxidant capacities. The results showed that the total antioxidant capacity of the four extracts determined by FRAP assay closely followed the extract sulfate content, indicating that this may be a variable of importance. However, this trend was not seen when antioxidant capacity was assessed using other methods [152], showing the conflicts in the literature on the effect of this variable. This conflict was furthered by Chen et al., who found that the anti-inflammatory and antioxidant activity of fucoidans increased with sulfate content while the anti-lipogenesis decreased, suggesting that the effect of sulfate content is unique on each biological activity [31]. A limitation of this study is that the sulfate content of the fucoidans used was artificially increased by a chemical modification. Therefore, the results of this study may not be representative of naturally occurring highly sulfated fucoidans. Similarly, Mandal et al. produced sulfated and desulfated forms of an extract through chemical means before testing on herpes simplex virus. The antiviral activity of the desulfated samples was significantly lower (IC50 >100 µg/mL) in comparison to that of the sulfated samples (19.5 µg/mL), suggesting increased antiviral activity [150].

Thuy et al. found no correlation between sulfate content and antiviral activity after producing extracts with increased sulfate content using anion-exchange chromatography [151]. This may suggest that the picture is more complex, and simply increasing sulfate content alone may not lead to an increase in bioactivity. Bioactivity effects may instead be related to the molecular conformation that allows for the sulfation of the polysaccharide structure [153]. Overall, the literature suggests that sulfate content does influence extract biological activity.

At present, fucoidan manufacturing should focus on producing extracts with a high purity and high molecular weight to allow for flexibility in the valorization and formulation of fucoidan into biologically active preparations. To achieve this, seaweed species and process variables must be optimized. However, further work is needed to understand the influence of other key quality attributes on different biological activities. For example, there is little literature on the effect of fucose contents. The effect of this variable may be explained by the monosaccharide being highly sulfated, with up to three sulfate functional groups [154]. As such, extracts with a high fucose content may have similar effects to those of extracts with a high sulfate content, and there would be a requirement to minimize other seaweed cell wall contents, including uronic acids, through purification for a potent bioactive extract. However, this should be investigated with more biological studies in order to elucidate the relationship between biological activity, fucoidan structure and quality attributes.

Fucoidan extract data reporting should be standardized to build up a large data set so that commonalities and trends can be spotted, e.g., by using machine learning tools to identify extract quality attributes required for a given bioactivity. Further emphasis should move from determining if an extract possesses a given bioactivity to mechanistic studies to understand how fucoidan acts on a specific microbial class. These studies would then allow for the required quality attributes to be identified and allow for greater specific discussions around individual bioactivities, e.g., antiviral and antibacterial, instead of general bioactivity.

### 6.3. Future Standardization Recommendations

One of the purposes of this review paper is to demonstrate that the standardized reporting of data on extract structure and contents would allow for a link between extraction techniques, extract quality attributes and, ultimately, bioactivity to be established. This was achieved by standardizing the large amount of information extracted from the literature using a set of assumptions. However, this methodology may mean that differences in the biological factors, extraction protocols and analytical methods employed are not captured fully. This could be attenuated if the methods of reporting were standardized.

As was established earlier in this review, both biological and process factors influence the final fucoidan product (Figure 1). These differences make the comparison of extracts between studies particularly challenging. In order to tackle the effect of biological factors, information on the raw seaweed used in an extraction should be provided whenever possible, including its species, harvest location, season and monosaccharide profile. For the process factors and the vast number of different pre-treatments, extraction and purification protocols employed, yields, molecular weight and contents should be reported between process stages so that the effectiveness of each can be assessed.

Another challenge in extract reporting is the vast number of analytical techniques employed, such as mass spectroscopy, anion-exchange chromatography and colorimetric methods, to determine key fucoidan quality attributes. An excellent overview of many of these methods is provided by Zayed et al. [22]. Colorimetric methods are widely used due to their simplicity and reduced cost. However, they also have associated error, which may lead to the over- or under-estimation of fucoidan content [155]. This limits their ability to be compared with liquid or gas chromatography methods. As such, to allow for their continued use, a well-characterized fucoidan standard should always be included so that comparisons can be made between studies.

Similarly, the way data is reported is fundamental. For example, yields are often expressed as a polysaccharide-rich (fucoidan, alginate, laminarin) extract yield relative to the amount of raw biomass used instead. This makes it difficult to compare key factors contributing toward the success of fucoidan extraction, especially when comparing fucoidan yields among different species. Efforts should be made to determine the fucoidan purity of such polysaccharide fractions so that it can be determined if they are actually fucoidan-rich extracts. Only then can an extraction method’s performance be truly assessed, and only then will it be possible to determine how different fucoidan yields are among different seaweed species. Finally, uronic acid contents are often reported together, but not all are from the same polysaccharide source. As such, a high uronic acid content is not necessarily an indicator of alginate contamination. Glucuronic and galacturonic acids can be part of branching sections of fucoidan, especially in fucoidan derived from Laminariales. Therefore, the individual uronic acid (glucuronic, galacturonic, glucuronic, etc.) content should be reported for accurate comparison.

Despite these challenges, this review made all efforts to show the performance variability of different methods by making assumptions to standardize extract data and allow direct comparisons to be made.

These comparisons suggest that all extraction methods offer similar performance in terms of yield, purity and contents. A notable result was that EAE produced extracts with a narrow range of molecular weights due to this method’s specific nature.

This crude way of standardizing the literature acts as a case study to demonstrate that if information on fucoidan extracts was uniform, comparison between methods would become easier.

The standardization of reporting for extraction contents and properties would allow for a more sophisticated form of this methodology to be applied and presents an opportunity of creating a large, detailed dataset to allow for deeper and more meaningful comparisons between extraction methods and conditions. A further possibility for the creation of this dataset could arise through use of machine learning tools, allowing for the relationships between extraction conditions and techniques, fucoidan contents, quality attributes and, ultimately, bioactivity to be determined.

## 7. Conclusions and Future Outlook

Numerous fucoidan extraction techniques have been developed over the years. These techniques employ a wide range of background technology, all with the same aim of breaking apart the seaweed cell wall to release its contents. Throughout this review, it is clear that a diverse number of pre-treatment, extraction and purifications protocols, each using a different seaweed source, are affected by their own unique sets of biological factors to produce a fucoidan extract. Afterward, the key quality attributes of each extract is analyzed using a number of different analytical techniques. This makes comparison between papers extremely challenging, and a true assessment of each extraction technique’s benefits is virtually impossible. To address this problem, the standardization of extract reporting is vital to aid in the development of knowledge surrounding specific extraction techniques and could help to unravel the relationship between extraction variables and fucoidan contents to aid in the selection of processing parameters for specific biological applications.

This review provides a template for standardization aiming to uncover the relationships between extraction techniques, extract characteristics (including extraction yield, molecular weight and total sugar, fucose, uronic acid, sulfate and polyphenol contents) and bioactive properties. This was achieved through a literature search to collect all existing data. The extracted data were then standardized through assumptions before collation (Table 1, Table 2, Table 3, Table 4 and Table 5 and Figure 5); this demonstrated that all extraction methods can produce fucoidan extracts with similar quality attributes (i.e., yield, purity and contents).

As such, a holistic approach for the selection of an appropriate extraction technique is required. A number of heuristics for the selection of an extraction method, considering the wider context, are provided within this review. Future work is required to continue developing knowledge within the suggested heuristic categories provided, e.g., technoeconomic analysis. Furthermore, a blueprint of best practices to produce high-quality fucoidan is provided.

Through an analysis of the literature, the key quality attributes required for a biologically active extract were suggested. Literature in this area should move away from discussing bioactivity in general and toward more specific effects, such as antibacterial properties. Mechanistic studies are also required to further develop knowledge on fucoidan.

## Figures and Tables

**Table 1:** Typical monosaccharide and uronic acid contents of the three main seaweed polysaccharides typically found in fucoidan extracts: fucoidan, alginate and laminarin. [17,47,48,49].

	Extract Contents	Polysaccharide Source		
		Fucoidan	Alginate	Laminarin
Monosaccharides	Fucose	✓		
	Galactose	✓		
	Xylose	✓		
	Mannose	✓		
	Rhamnose	✓		
	Glucose	✓		✓
	Mannitol			✓
Uronic Acids	Glucuronic acid	✓		
	Galacturonic acid	✓		
	Guluronic acid		✓	
	Mannuronic acid		✓	

## Data Availability

Not applicable.

## Abbreviations

USD	United States dollar
MAE	Microwave-assisted extraction
UAE	Ultrasound-assisted extraction
EAE	Enzyme-assisted extraction
PLE	Pressurized liquid extraction
HPAEC-PAD	High-performance anion-exchange chromatography with pulsed amperometric detection
T	Extraction temperature
t	Extraction time
v_SL_	Solid-to-liquid ratio
[E]	Enzyme concentration
P	Microwave/ultrasound power
T_u_	Ultrasound extraction time
I	Ultrasound extraction current
A	Ultrasound extraction amplitude
ƒ	Ultrasound extraction frequency
t_m_	Microwave extraction time
P_i_	Pressurized liquid extraction pressure
t_p_	Pressurized extraction time
n_cycles_ MW	Number of pressurized liquid extraction cycles Extract molecular weight
DI H_2_O	Deionized water
H_2_O	Water
NaOAc	Sodium acetate buffer
HCl	Hydrochloric acid
Tris-MES	Tris(hydroxymethyl)aminomethane (2-(N-morpholino)ethanesulfonic acid buffer
MgCl_2_	Magnesium chloride
EDTA	Ethylenediaminetetraacetic acid
ChCl-Gly	Choline Chloride-Glycerol
NaN_3_	Sodium azide
H_2_SO_4_	Sulfuric acid
NaOH	Sodium hydroxide
HCOOH	Methanoic acid
EtOH	Ethanol
